# High-Performance SiC–Based Solar Receivers for CSP: Component Manufacturing and Joining

**DOI:** 10.3390/ma14164687

**Published:** 2021-08-19

**Authors:** Valentina Casalegno, Luca Ferrari, Maria Jimenez Fuentes, Alessandro De Zanet, Sandro Gianella, Monica Ferraris, Victor M. Candelario

**Affiliations:** 1 Department of Applied Science and Technology, Politecnico di Torino DISAT, 10129 Torino, Italy; alessandro.dezanet@polito.it (A.D.Z.); monica.ferraris@polito.it (M.F.); 2 EngiCer SA, 6828 Balerna, Switzerland; luca@engicer.com (L.F.); sandro@engicer.com (S.G.); 3 Department of Research and Development, LiqTech Ceramics A/S, 2750 Ballerup, Denmark; mjf@liqtech.com (M.J.F.); vcl@liqtech.com (V.M.C.)

**Keywords:** SiC, SiSiC, CSP, joining, ceramic foam

## Abstract

Concentrated solar power (CSP) is an important option as a competitive, secure, and sustainable energy system. At the moment, cost-effective solutions are required for a wider-scale deployment of the CSP technology: in particular, the industrial exploitation of CSP has been so far hindered by limitations in the materials used for the central receiver—a key component in the system. In this context, the H2020 NEXTOWER project is focused on next-generation CSP technologies, particularly on advanced materials for high temperatures (e.g., >900 °C) and extreme applications environments (e.g., corrosive). The research activity described in this paper is focused on two industrial solutions for new SiC ceramic receivers for high thermal gradient continued operations: porous SiC and silicon-infiltrated silicon carbide ceramics (SiSiC). The new receivers should be mechanically tough and highly thermally conductive. This paper presents the activity related to the manufacturing of these components, their joining, and characterization.

## 1. Introduction

Concentrated solar power (CSP) is a technology that generates electricity by using thermal energy from solar radiation, which is focused on a small area. Solar radiation coming from the sun is reflected by a large area of mirrors onto the small area (receiver), where it is converted to heat. This, in turn, is used to produce steam, which drives a generator to produce electricity.

The concentration of solar radiation on the small area of the receiver enables the achievement of high temperatures (ranging from 400 °C to 1000 °C) of the working fluid, thus making the CSP technology thermodynamically comparable with conventional power plants.

The objective of the H2020 NEXTOWER project is the development of innovative materials to boost the performance of solar power towers and innovative technologies for energy storage to improve performance, life cycle, and competitiveness of concentrated solar power plants [1].

The industrial exploitation of CSP power plants has been so far hindered by limitations in the materials used for the solar collector (more specifically for the receiver, i.e., the core component) and for thermal storage [2].

The solar collector is the main device in CSP for taking advantage of solar energy to achieve various targets. It is composed of a reflector and a receiver; in particular, the performance of the whole system strictly depends on the receiver material.

A volumetric receiver consists of a porous component; it produces the so-called volumetric effect, which means that the irradiated side of the absorber is at a lower temperature than the medium leaving the absorber. The porous structure acts as a convective heat exchanger where the heat transfer fluid (i.e., air) is imposed to absorb the solar radiation by convection heat transfer mode.

Past experience [2,3] emphasized difficulties and prematurely failures in operating air-based CSP tower systems for an extended time, suggesting that the primary limiting factor was the solar receiver.

In particular, the choice of the materials for the solar receiver manufacturing affects several factors, such as temperature range, plant capacity, and heat transfer properties, as stated in [4]. At the same time, manufacturing technologies (including joining processes) play important roles in achieving the desired efficiency.

The state of the art for atmospheric volumetric receivers for large solar towers has been extensively reviewed in [5]. Two different solutions have been reported: the first was based on wire-mesh metallic absorbers and the second one on SiC ceramic air channeled structure.

The wire-mesh metallic absorbers allow limited air temperature up to 550 °C and present high oxidation rates on the absorber material; the SiC-based one can operate up to 850 °C and allows the receiver to work under higher radiation.

The use of a ceramic structure can meet the thermal requirements as well as improved mechanical properties such as higher bending and compressive strength; a promising ceramic structure for the volumetric receiver can be achieved by proper tuning of porosity, pore size distribution, and microstructure of the ceramic material.

In particular, the requirements of a volumetric receiver can be summarized as follows:

- Thermophysical and optical requirements (absorption, heat transfer surface, high fluxes, and radial heat transport);
- Material requirements (high porosity, high cell density, dark, thermal conductivity, 3D structure, high thermal shock resistance, high melting point, longer material lifetime, and stability at high temperatures).

Some authors [6,7,8] report on the porous microstructure of the volumetric solar receivers; the use of porous ceramic material can offer several advantages as those above listed, for instance, thin walls, high geometric surface area and, as a consequence, good gas–solid contact, accommodation of high gas flow rates combined with low-pressure drop and good mass transfer performance.

Among the class of ceramic materials, SiC-based ceramics show superior thermal properties: the enhanced absorbance of SiC due to its natural black color coupled with its high thermal conductivity allows the collection of solar heat and the effective heating of the medium inside the channels of the porous structure.

Even though ceramic materials show promising behavior in terms of thermal performance, a sudden mechanical failure from severe thermal cycling represented their limiting factor.

In fact, it has been reported [5] that the failure in the volumetric receiver usually occurs in two critical points: the joining between tile and cups and in the cups themselves.

According to what was discussed above, manufacturing of new CSP materials within the H2020 NEXTOWER project was focused on porous SiC ceramics for high-temperature receivers, characterized by high thermal shock and high thermal fatigue resistance and capable of extended service operations for 20–25 years; in particular, the volumetric solar receivers described in this paper are based on SiC ceramic porous structures. The use of SiC porous structures should improve thermal losses and increase the transmission of tubular receivers [9,10,11].

The NEXTOWER solar receiver is formed by many single units, each one made of three ceramic parts: a tile, a Venturi-shaped cup, and a cup support; the tile and the cup are joined together.

In this paper, a focus on the materials’ performances for the solar receiver is reported, based on the consideration that the maximum working temperature and the overall in-service durability depends on the materials’ performances.

The novelty of the present research activity regards the design and manufacturing of new SiC cellular structures to re-design the solar receiver.

The new proposed receiver has not been realized and tested before, and it is supposed to achieve unparalleled service life at a much higher temperature than previous ones; this is the main added value of this paper.

The specific objective of this study regards industrial solutions for new SiC ceramic receivers for high thermal gradient continued operations; porous SiC and silicon-infiltrated silicon carbide ceramics (SiSiC) have been manufactured, taking into account the requirements of toughness and high thermal conductivity of the final component, as well as the assembly issues characteristic of these materials.

The manufacturing processes and the joining materials and techniques developed for receivers described hereafter have the following working targets: extreme thermal cycling (gradients of max 70 °C/cm) without failure at a maximum material temperature of at least 800 °C, lifetime over 20 years of continuous operation.

Two industrial-grade solutions, based on two proprietary technologies by two European companies LiqTech Ceramics A/S (Ballerup, Denmark) and EngiCer (Balerna, Switzerland), were selected for the manufacturing of the solar receivers:

(1) Innovative high-temperature open volumetric receivers based on all-SiC honeycomb design (hereafter called “all-SiC”) for increased durability and oxidation; LiqTech own technology provides a strong and high thermally conductive SiC honeycomb structure based on commercial SiC filters for diesel engines. The design has been adapted to create a recrystallized SiC monolithic component by a SiC-based joining at temperature above 2000 °C.
(2) Joined receivers based on silicon-infiltrated silicon carbide ceramics (SiSiC) in the shape of periodic cellular structures (hereafter called “SiSiC”) optimized for higher toughness and thermal conductivity. EngiCer provides silicon infiltrated SiC-based lattice structures produced by special rapid prototyping technology that allows screening and optimizing the geometry for the specific application within CSP plants.
  A specific joining process has been designed and carried out for SiSiC joined receivers by Politecnico di Torino (Italy).

## 2. Materials and Methods

### 2.1. All-SiC

The highly porous All-SiC solutions were manufactured by LiqTech Ceramics A/S.

All-SiC product is based on partially sintered highly porous recrystallized SiC, and it is manufactured in two parts; the first part is the “tile”, which is produced by extrusion, and the second part is the “cup”, which is fabricated by slip casting in a gypsum mold. Both parts are produced separately and then joined together with SiC paste and then sintered at a temperature above 2000 °C; after firing, the solar receiver is slightly oxidized to remove the free carbon on its surface.

For the manufacture of the tiles and cups, a mass or paste should be developed with similar composition.

Two different α-SiC powder batches were used, with a particle size between 20 and 35 µm for the coarse one and between 0.4 and 0.8 µm for the fine one; this different powder granulometry of the SiC powders is necessary in order to achieve the right recrystallization mechanism during sintering [12,13].

The paste (or slurry) was prepared by using the fine and coarse SiC powders, together with a plasticizer (to give the right plasticity to the mass and paste), a binder, dispersant, and water and ethanol as solvents.

The tiles are manufactured by doing an extrusion slurry that consists of a very high solid load, between 70 and 80 wt% solid. The extrusion slurry is stirred for 1 h and then put in the extrusion equipment to produce the desired shape of the tiles with an extrusion pressure of about 30 bars. The final shape is given by cutting.

The cups are produced by a slip casting slurry that has lower solid loading than the extrusion mass (in the range of 60–70 wt%); the slurry is poured in a gypsum mold for a certain time.

Once the tiles and cups are dried for 48 h, they are joined as green parts with a SiC paste with a similar composition of the paste for the fabrication of the tiles and cups and let to dry for 24 h.

To find the optimal sintering temperature to obtain high mechanical resistance, samples (in the green state) are sintered at three different temperatures, chosen in the range to achieve SiC sintering (2000 °C < T < 2400 °C, temperature determined using a pyrometer), for 1.5 h under argon atmosphere. Finally, the sintered samples are treated in an air furnace (ELS 1000 S SOB, Helmut Rohde GmbH, Prutting, Germany) to remove the free carbon in the pores.

**Figure 1:**
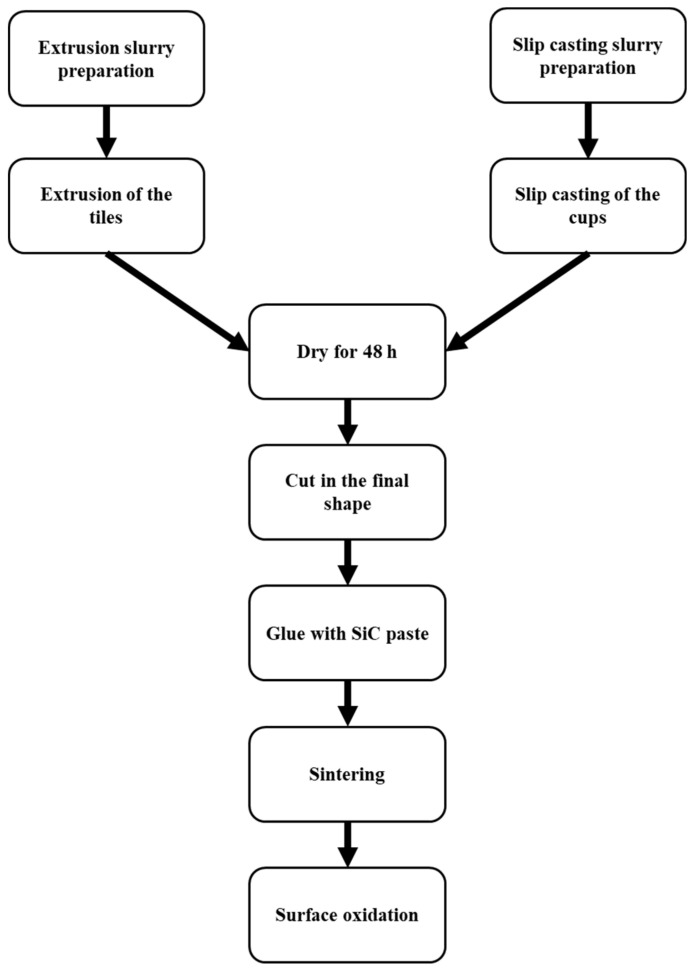
Schematic representation of the process flow for all-SiC solar receivers.

Figure 1 shows a schematic representation of the process flow to manufacture all-SiC ceramic solar receivers.

Tiles and cups were characterized by Scanning electron microscope, FlexSEM 1000 (Hitachi GmbH, Stockholm, Sweden) and Hg porosimetry (Poremaster© GT, Quantachrome Instruments, Leipzing, Germany) to check their stability.

Symmetrical four-point bending tests were performed at RT according to the C1674-16 standard [14]. The four-point bending test was performed using a universal machine (Testometric M350-5CT, Rochdale, UK), with a crosshead speed of 5 mm/min with inner and outer spans of 40 mm and 80 mm, respectively.

At least 15 samples were tested for each batch.

### 2.2. SiSiC

Silicon-infiltrated silicon carbide ceramics (SiSiC) for solar receivers were designed and manufactured by EngiCer. SiSiC are used for a wide range of engineering applications due to their excellent near-net-shape fabrication and good mechanical properties coupled with high chemical stability up to elevated temperatures.

For the manufacturing of SiSiC solar receivers, a lattice geometry was designed, dimensioned and printed through additive manufacturing in Nylon PA12. The morphology chosen for the prototypes has been optimized and experimentally validated to achieve optimal thermal efficiency and mechanical stability.

The printed polymeric models served as template structures for the ceramization process, and they give the final prototype the desired morphology. The ceramization process employed to manufacture the sample is called the *replica* technique [15] (Schwarzwalder method), which consists of the impregnation of a sacrificial polymeric template with a SiC-based ceramic slurry. The coated template structure, also called the green body, is then dried, pyrolysed in an inert atmosphere up to 1000 °C, and liquid silicon infiltrated at 1500 °C.

**Figure 2:**
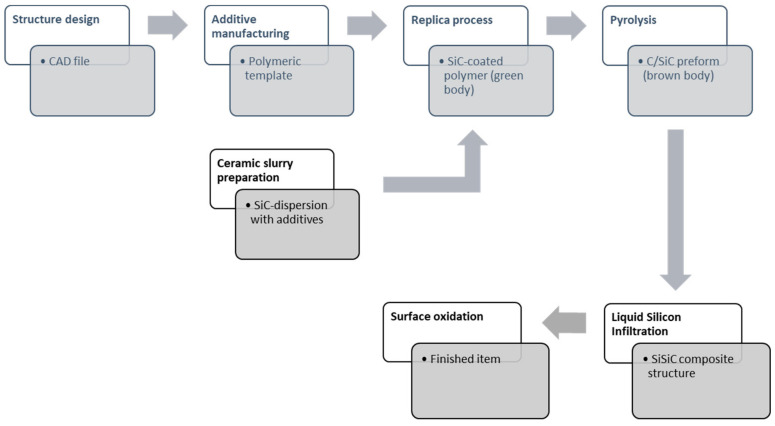
Process sketch for SiSiC lattices manufacturing.

The optimization process for the SiSiC component manufacturing takes into account the decomposition process of the green body that leads to a skeleton material with a certain desired porosity. This porosity allows liquid silicon to fill the microstructure via capillary forces and, at the same time, produce a final fully dense lattice geometry suitable for solar receiver application. The last step in the production of SiSiC for this special application is a thermal cycle at 1500 °C in air. The cycle serves to oxidize the lattice surface to reduce the reflected radiation. A schematic representation of the process flow for the manufacturing of SiSiC lattices is given in Figure 2.

### 2.3. Joining Process

**Figure 3:**
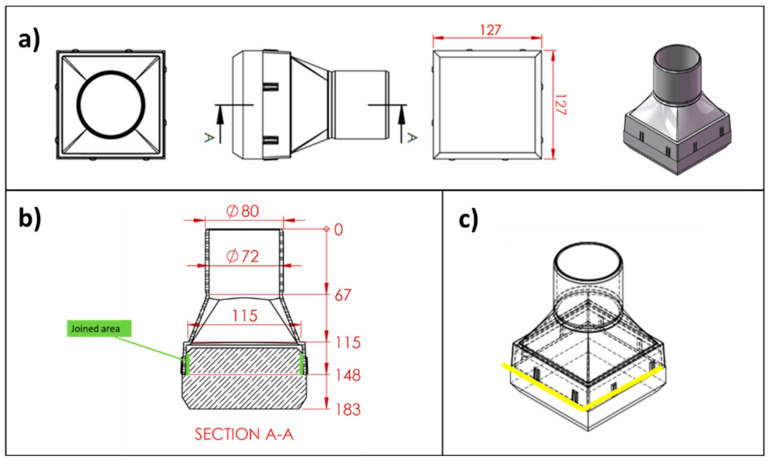
Sketch of cup/foam joint; (**a**) the tile and the Venturi-shaped cup; (**b**) section A-A: joined areas are indicated in green colour; (**c**) schematic of possible joining design on two sides of the cup (yellow colour) instead of four. Dimensions are in millimeters.

The joining process was developed to join the SiSiC lattices (or foam) and the SiC-based cups (according to Figure 3a,b).

The joining technique called “RM-wrap” (RM = refractory metal) was developed and characterized in previous studies [16,17]; it allows to obtain refractory-metals disilicides based joints using a pressureless method at 1450 °C.

In the experimental activity described in this paper, molybdenum was used as refractory metal in this joining material.

Materials to be joined (flat surfaces and foam) were cut, sonicated in ethanol in order to remove dirt and dust, dried in air, and then joined.

The Mo-wrap method consists of wrapping Si foils inside a Mo wrap to prevent molten silicon leak from the joined area and infiltration in the SiC foam and SiC-based substrate during the joining process.

Several joined samples of about 10 mm × 10 mm × 10 mm were prepared and used for microstructural investigation.

Larger mock-up samples of about 40 mm × 40 mm × 30 mm were made with the same method, i.e., by joining a SiSiC lattice of about 40 mm × 40 mm × 40 mm to an “L”-shaped SiC component by a SiSiC pin used as a mechanical fastener.

**Figure 4:**
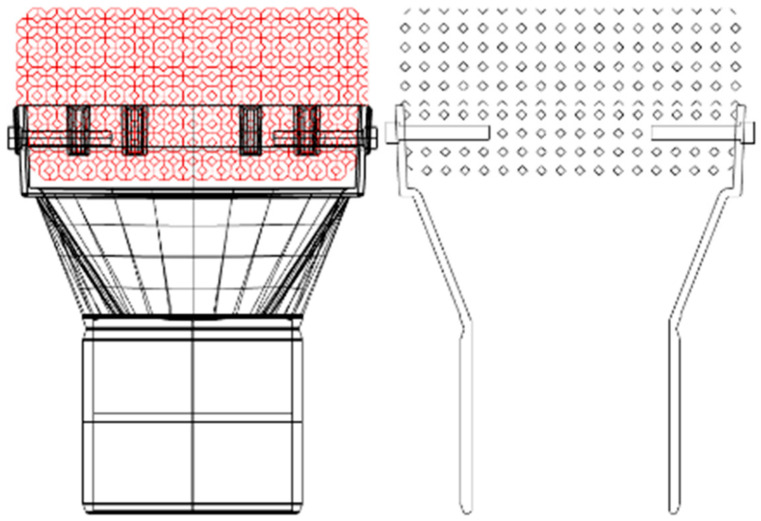
Sketch of the solar receiver mock-up; a SiSiC pin is used together with the Mo-wrap joining to increase mechanical strength.

The design of this mock-up is shown in Figure 4. The foam was machined to obtain a hole where the ceramic pin can be inserted; a narrow tolerance between the pin diameter and the hole diameter has been taken into account for the placing of the Mo-wrap joining material.

The molybdenum (Alfa Aesar Germany, 25.4 microns thick, 99.95% Mo) and silicon foils (MEMC Electronic Materials, S.p.A, Novara, Italy, 584 microns thick, 99.95% Si) were used as joining materials. The joints were processed in a tubular furnace (BICASA, Bernareggio, Italy) under Argon flow (Ar T, Ar 99.9995%) at 1450 °C with a Ti sponge as an oxygen getter, dwelling time 5 min, heating rate of 1000 °C/h, without applying any pressure. The joining material was obtained by wrapping one Si foil in a Mo foil folded as a wrap.

The surface morphologies of SiC-based materials and the microstructure of the joined sample were characterized by a scanning electron microscope (QUANTA INSPECT 200, Zeiss SUPRA TM 40) coupled with an energy dispersive X-ray spectroscope (EDAX PV 9900). The reaction phases formed in the joints were identified with an X-ray diffractometer (XRD, Philips X’Pert) using the X’Pert HighScore Plus program for phase identification (results not reported here).

The 3D foam structure joined to SiC substrate was investigated by micro-CT.

For this purpose, a Phoenix v|tome|x m 300 instrument (X-ray microfocus CT system for 3D metrology and analysis with up to 300 kV) was used; the samples were scanned by X-ray tomography using the following parameters: (i) 270 kV di filament voltage; (ii) 125 µA filament current; (iii) 500 ms exposure time per projection; (iv) 1500 projections over 360° rotation; and (v) pre-filters: Sn, thickness 0.5 mm, Cu, 0.5 mm thickness.

The tomography was registered by 3D translation and rotations, and digital volume correlation (30 µm/voxel resolution) was applied to measure the relative 3D displacement fields.

## 3. Results and Discussion

The goal of the experimental activity was to develop independently and simultaneously two designs to allow a comparison of all-SiC design optimized for oxidation vs. the SiSiC design optimized for higher toughness and thermal conductivity.

Further activity will be addressed to test the manufactured SiC-based solar receiver in a solar power plant.

### 3.1. All-SiC

The solid content of the extrusion and slip casting slurry plays a crucial role in the fabrication of the all-SiC solar receivers. In the case of the extrusion slurry, the solid loading ranges between 70 and 80 wt% in order to achieve a defect-free monolith.

A specific study changing the solvent content was done, together with the monitoring of the humidity of the material and the extrusion pressure.

According to this experimental activity, an optimized solid loading content was used for the slurry, having a humidity of 11.7 %. Defect-free and homogeneous tiles were produced using extrusion pressure of 30 bars [12,18].

**Figure 5:**
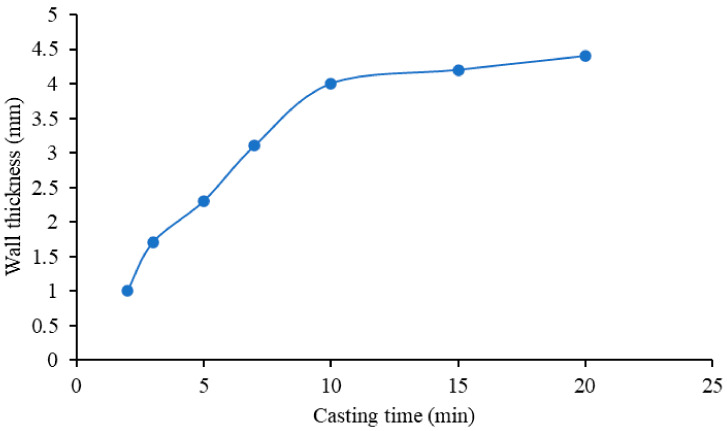
Wall thickness as a function of the casting time for the cups’ production.

With respect to the cups, two parameters must be taken into account to obtain a homogeneous monolith: the solid content and casting time. The first parameter was kept stable during all the experiments in the range from 60 to 70 wt%. With regards to the second parameter, different casting times were used to obtain a defect-free component with optimized wall thickness. Figure 5 shows an average value of the wall thickness with respect to the casting time, measured for five different samples for each casting time. The wall thickness increases with the increasing casting time until a certain time when the mold is saturated, and the wall thickness cannot grow anymore [19,20] because, in drain casting, the cast grows proportionally to the square root of the casting time, i.e., capillarity suction of water into the gypsum mold, forming and growing the cast. The wall thickness also indicated as cast thickness *D*, as a function of the casting time *t* is given the approximate equation [21], where *J* is the volume of cast/volume of liquid removed, Δ*P* the apparent mold suction, *K_p_* the liquid permeability of the cast, and *µ* the viscosity of the liquid transported.

$$D≅{\left(\frac{2J\Delta PK_{p}}{\mu }t\right)}^{0.5}$$

The optimal casting time for the cups was proven to be 10 min, with a layer thickness of 4 mm (standard deviation ± 0.4 mm) and a defect-free and homogeneous monolith. It is important to find a relationship between the layer thickness and the production time consuming; a layer thickness of 4 mm is sufficient to have a strong cup, and it does not lead to time-consuming casting processes.

Once the tiles and the cups were dried and cut in the final shape and sizes, both parts were glued with a SiC paste in green.

**Figure 6:**
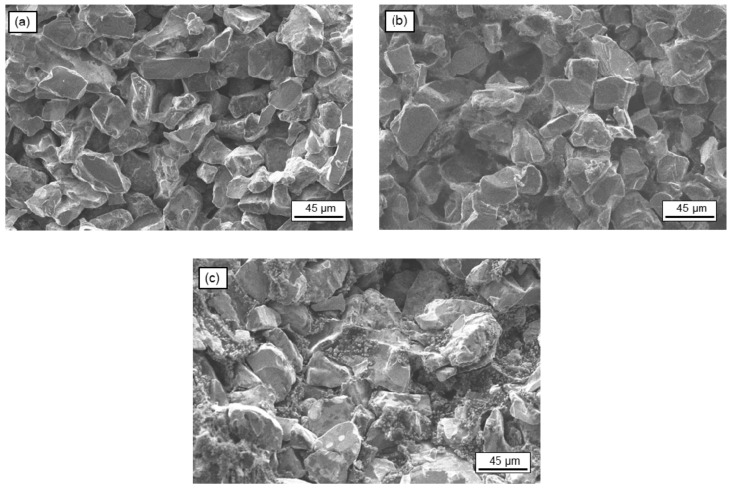
SEM images of the cross-section of the monoliths as a function of the sintering temperatures: (**a**) at T °C, (**b**) at T-100 °C, and (**c**) at T-200 °C. (T °C—the highest sintering temperature, confidentiality reason).

The final green monolith was sintered at three different temperatures, in the range of 2000 °C < T < 2400 °C, for 1.5 h under argon atmosphere. The cross-section morphologies with respect to the sintering temperature are summarized in Figure 6. For confidentiality reasons, hereafter, the highest sintering temperature will be referred to as T °C, whereas the other two temperatures will be referred to as T-100 °C and T-200 °C, respectively.

When the solar receiver was sintered at the highest temperature of T °C (Figure 6a), the connection between the grains was clearly visible, as well as the disappearance of the small particles in favor of the large particles’ growth. At this temperature, the surface diffusion and evaporation/condensation mechanism took place due to the atoms on the surface of the fine powders and edge parts of the coarse particles diffused to form neck regions between the coarse particles.

When the sintering temperature was decreased to T-100 °C (Figure 6b), no significant differences in the cross-section were observed in terms of the junction of the grains. At this temperature, surface diffusion and evaporation/condensation mechanism were predominant as well and presented high quality as with the previous temperature.

When the sintering temperature decreased even more to T-200 °C (Figure 6c), the disappearance of the fine particles was not observed. The process temperature was not sufficient to interconnect the small particles together to become relatively larger on the surface. Reduced microstructural coarsening was observed because of lower sintering temperature.

Figure 6 shows that when the sintering temperature decreases, there is less microstructural coarsening, and the recrystallization of the SiC is not complete. In all the cases, it can be observed that the particles remained equiaxed as expected for SiC materials due to the stability of α-SiC at high temperatures [12,22].

To evaluate the mechanical strength of the monolith, a 4-point flexural test was performed to corroborate the conclusion made with the SEM images. Since this test is a destructive test, an additional monolith with the size of 10 × 20 × 100 mm was manufactured for each production batch in the same sintering furnace.

The strength of the monoliths was 55 ± 2.5 MPa, 54 ± 1.3 MPa, and 42 ± 3.7 MPa for temperatures of T, T-100, and T-200 °C, respectively. Samples processed at T and T-100 °C showed similar mechanical strength values; because of the low temperature involved and the not complete sintering of the material, the samples sintered at T-200 °C had lower mechanical strength [23].

In order to remove the residual carbon that may remain in the pores, a surface oxidation step was carried out at a temperature of 1100 °C in air.

**Figure 7:**
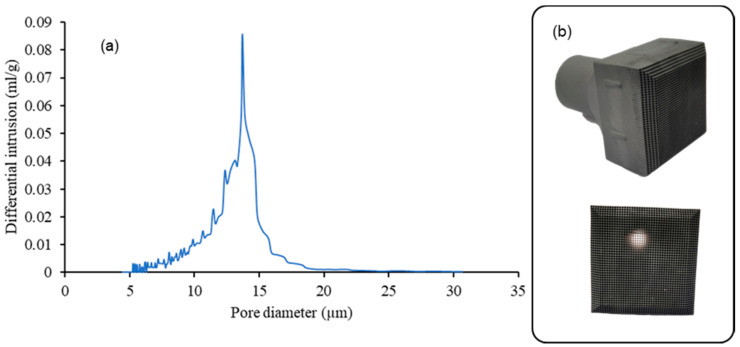
(**a**) Hg porosimetry of the monolith at T-100 °C, (**b**) picture of an example of the final product.

To summarize, the optimal temperature chosen for the fabrication of all-SiC solar receivers is T-100 °C, due to higher mechanical strength and the good joining of the particles. The range of porosity of this monolith is 42–45%. Figure 7 shows the final all-SiC solar receivers and their pore size measured by Hg porosimetry, with D50 of pore size of 17 µm.

### 3.2. SiSiC

**Figure 8:**
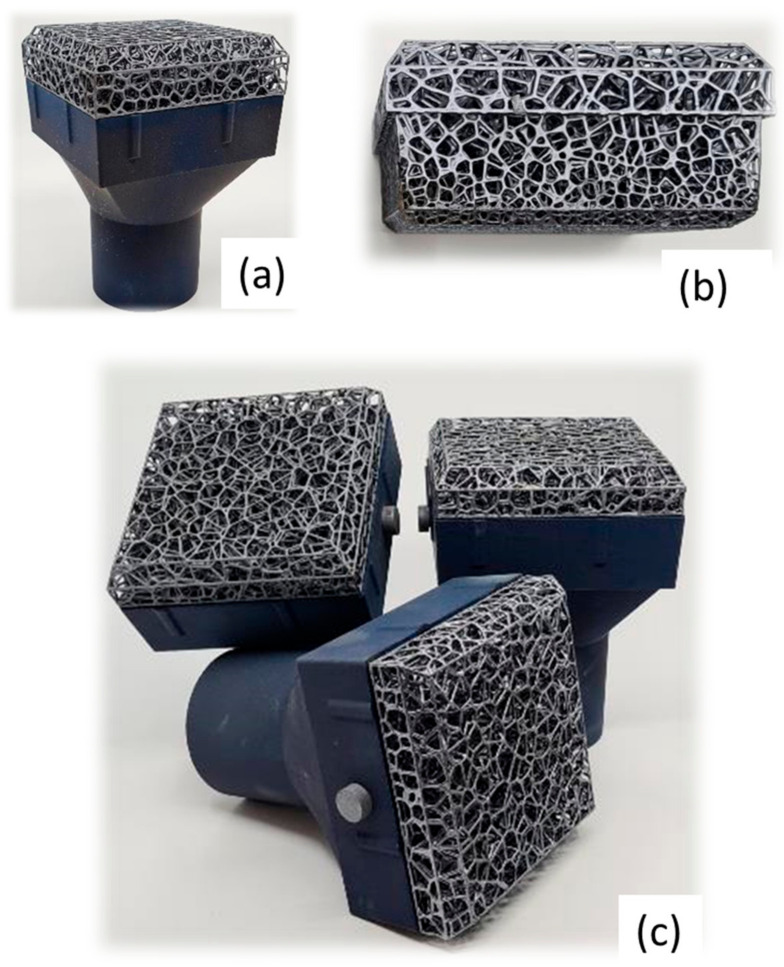
(**a**) SiSiC solar receiver designed and produced by EngiCer; (**b**) macrograph of the SiSiC lattices (**c**) SiSiC-SiC (foam and cup) assembled with the pin.

A SiSiC structure with Voronoi geometry was designed and manufactured based on lab-scale efficiency measurements. A porosity gradient was introduced in the flow direction to increase the heat exchange efficiency. The range of porosity of this structure is 83–85% on the face with small pores (inside part) and became 91–92% on the opposite side with big pores (outside part). The specific surface area is 0.42 [mm^2^/mm^3^]. Heat treatment was carried out on the produced SiSiC item with the aim of obtaining a matt-finished surface with the consequent reduction of the reflected radiation (Figure 8).

The relevant heat transfer properties depend on the porosity and pore size (or pore density). In the pursuit of achieving the volumetric effect and increasing the thermal efficiency of the receiver, numerous numerical and experimental studies have been undertaken to study the impact of porosity and pore size on the heat transfer; further results will be addressed by post mortem analysis of SiSiC components to be tested in the solar furnace at the Plataforma Solar de Almería (PSA), Almeria, Spain.

A test bench for the evaluation of volumetric ceramic components has been designed and put into operation for the solar thermal ageing test of SiC volumetric receivers in the SF60 Solar Furnace, Plataforma Solar de Almería (PSA), a dependency of the Centro de Investigaciones Energéticas, Medioambientales y Tecnológicas (CIEMAT), the largest concentrating solar technology research, development and test center in Europe. Different volumetric cups, provided by Liqtech and EngiCer, have been aged under concentrated solar radiation and open-air conditions, reaching 900 °C in the outlet air, in order to select the most promising materials and configurations to be tested in the Solar Central Receiver facility, CESA I (Spain). Work is in progress.

### 3.3. Joining Process

**Figure 9:**
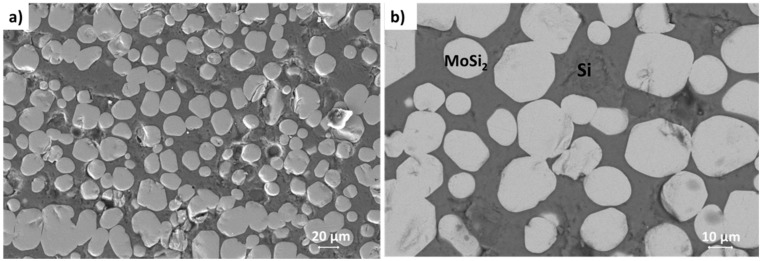
Microstructure of the MoSi_2_/Si joining material; it is an in-situ formed composites/MoSi_2_ particles embedded in a Si matrix), (**a**) cross-section of the joining material; (**b**) higher magnification micrograph: MoSi_2_ particles are round shaped and their size (diameter) ranges from 10 to 40 μm.

The joining materials used in this work have a composition ranging 32–35 weight % Mo and 65–68 weight % of Si. The microstructure of the resulting joining material is a silicon matrix with MoSi_2_ particles embedded in it, as shown in Figure 9.

The joint thickness ranges within 230 ± 30 μm, measured ex-post joining; the thickness was measured on 10 samples on the polished cross-section after cutting them. No significant variation in the thickness was observed in the joint, all along the joined surface area. It may be concluded that there was no edge effect due to not homogeneous distribution of joining material.

This joining technique has been already successfully used on several SiC materials [24,25], with several surface features and finishing; it allowed to join both dense and smooth surfaces (as CVD-SIC) and porous structures, as lattices and foam.

**Figure 10:**
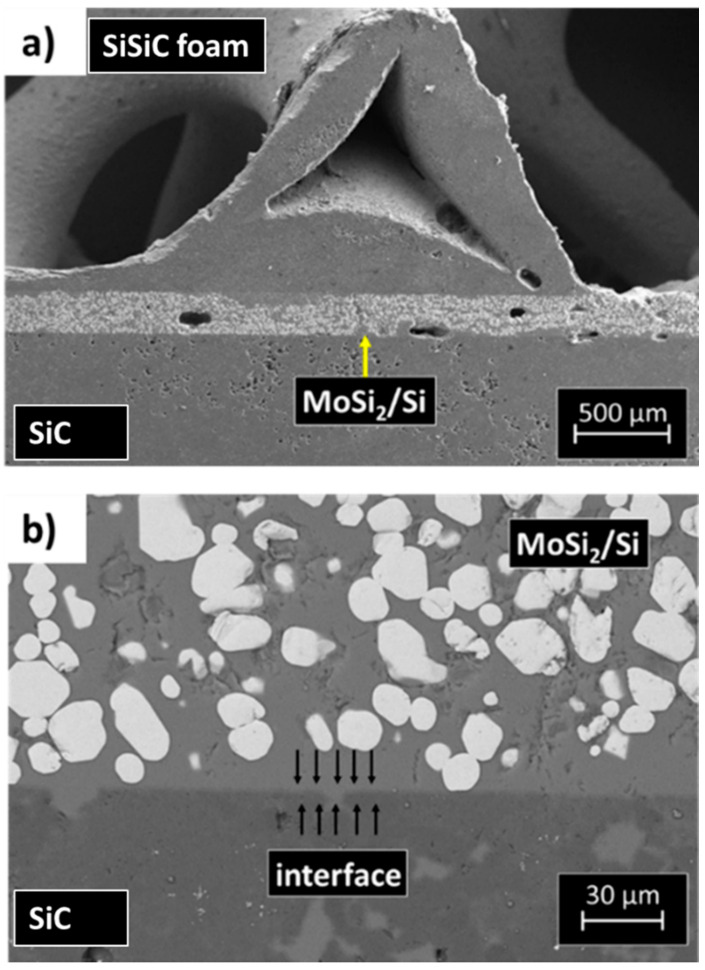
SEM cross-sections of a SiC-SiSiC foam sandwich (**a**) prepared by means of the Mo-Wrap joining technique; magnification of the substrate/joining material interface (**b**).

As an example, the microstructure of a joint manufactured using the Mo-wrap method is shown in Figure 10; the joining material (MoSi_2_/Si composite) is well distinguishable in Figure 10b. The highly porous SiC foam is joined to the SiC based skin in few connecting points. The foam and the substrate used for this activity have been supplied by EngiCer, showing features described in Section 2.2.

As already discussed in [24], the infiltration of the joining material in the foam porosity is an issue because of the high temperature (above silicon melting point) involved in the joining process.

At the same time, there are very few contact points between the foam and the substrate, and the contact interfacial area is very small. Adjusting the silicon infiltration and, at the same time, manufacturing strong joints is quite challenging, and an accurate study to optimize the process and to fully characterize the joints has been carried out.

A varying size of joining material gap restricts the uniform distribution of the brazing alloy (i.e., Mo-Si based material) within the joint. The capillarity of the gap is much reduced or does not occur in the immediate vicinity of the porosity of the foam.

The manufacturing of a larger sample showed a criticality, at least for the processing parameters transfer to larger surfaces and bigger furnaces. Moreover, the upscaling of the process requires accurate control of the joining material amount. In addition, applying the brazing materials on all four walls of the cup (i.e., on the walls of the squared base of the SiC cup) could lead to residual stresses all along with the joined interface (Figure 3).

In order to define the final design of the SiSiC receiver, it was decided to perform the joining process only on two sides of the cup instead of four (Figure 3c); it is proven to be useful also in terms of tolerance.

Some joined samples have been manufactured according to the above described two sides design, but the upscaling of the process, i.e., the transfer from lab scale to the industrial scale, evidenced some issues. More in detail, the use of bigger furnaces to perform the joining process can hinder the precise control of joining temperature; in the case of the Mo-wrap joining process, the temperature exceeds the Si melting point and must be accurately controlled.

The heating must be homogeneous in order to avoid silicon leakage during its melting and/or overheating of some regions. Moreover, the manufacturing of larger samples is critical in terms of homogeneous distribution of joining material: the wrap must be calibrated to produce the in-situ composite joining material with a reproducible distribution of the second phase (MoSi_2_ particles) in the Si matrix. Additionally, a border effect can occur, and it severely influences joining morphology for larger samples and, as a consequence, their thermomechanical behavior.

In order to overcome these issues and to increase the mechanical strength of the joined parts (foam and substrate), it was then decided to use a hybrid Mo-wrap/pin technology to obtain the upscaled sample. The Mo-wrap can improve the mechanical resistance of the pin-based solution, can reduce the Mo-wrap joined area, thus avoiding up-scale related issues, and finally provide a final component with better properties than those initially foreseen for the Mo-wrap based prototype (Figure 4).

On the other hand, mechanical fastening can add materials (i.e., the pin) and production cost to the assembly; the pin is manufactured in SiSiC, and its weight can be considered negligible compared to the final component. The positioning of the pin in the hole to obtain the mechanical joint can be a critical issue, but it can be mitigated by optimizing the amount of joining material close to the pin.

**Figure 11:**
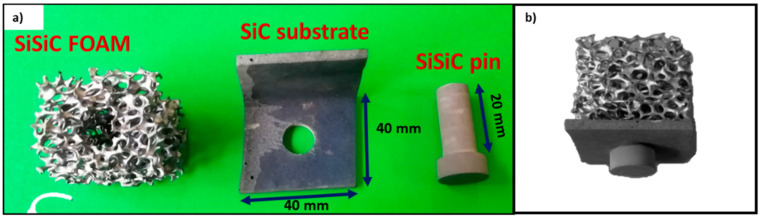
Parts of the mock-up of the solar receiver: the SiSiC foam, the “L” shaped SiC substrate, the SiSiC pin (**a**), and the joined mock-up (**b**).

The parts to be assembled for obtaining the solar receiver mock-up are shown in Figure 11a. The idea is to manufacture a prototype representative of the final component shown in Figure 4.

Several joining configurations were tested to optimize the joining process. Best results were obtained by putting the joining material (as Mo-Si wrap) on the top of the pin and on the bottom surface of the “L” shaped SiC substrate. The joining material can also be positioned on the vertical side of the “L” shaped SiC substrate; experimental work is in progress to perform this joining solution, even if some criticalities has been encountered.

**Figure 12:**
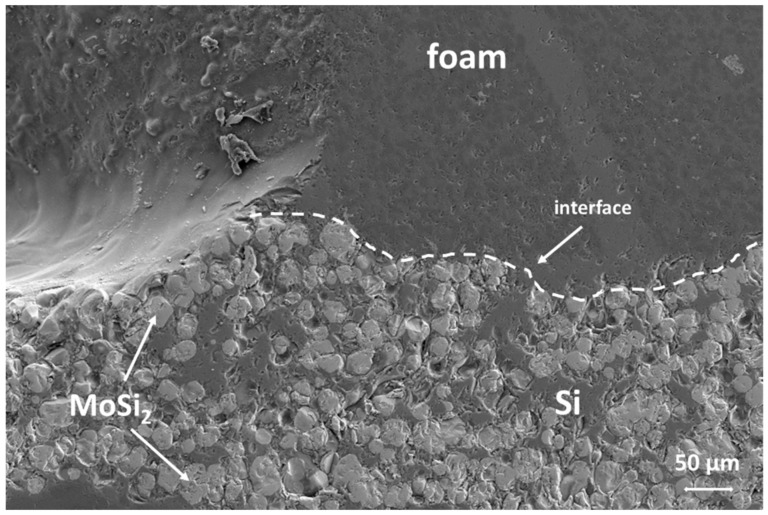
SEM image (BSE) of the interface between the foam and the joining material; on the left, a pore in the foam structure can be detected, while a magnification of a foam leg connected to the joining material is visible on the right.

Figure 11b shows the obtained mock-up with the optimized set-up. It was observed by SEM analysis (Figure 12); the cross-section of the joined area, i.e., at the interface between the foam and the “L” shaped SiC and at the interface between the foam and the pin, shows a rather continuous interface. The foam ligaments are embedded in the joining material. Critical points can be identified where the foam legs are not in contact with the pin or the substrate (due to random length of foam legs around the hole manufactured for inserting the pin), or there is a gap that cannot be filled by the thickness of the joining seam.

The observed morphology of the joining material is identical to the one reported for smaller samples: it is worth pointing out that this is an important result since the joining process upscaling can be an issue, and more specifically in the case of joining material as a wrap.

**Figure 13:**
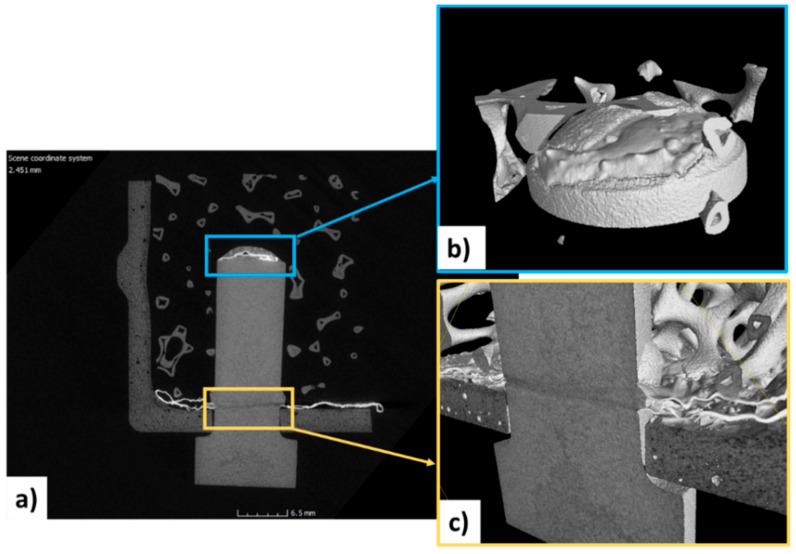
Tomographic reconstruction of the joined mock-up: cross-section (**a**) details of the joining material on the pinhead (**b**) and near the hole (**c**).

The micro-CT (Figure 13) allowed the joined interfaces to be clearly visualized and to detect cracks or pores or lack of joining material derived from the partially unsuccessful joining process. The soundness of the joints has been confirmed, and no cracks have been detected both in the joining material and in the joined components.

Micro-CT analysis permitted the determination of other important microstructural features: the joining material remains on the surfaces where it was placed before the joining process. The flowing of molten silicon is avoided by the Mo-wrap configuration; in fact, on the vertical wall of the pin, no joining material is detected. It confirmed that the joining material placed on the top of the pin remained in the same position.

**Figure 14:**
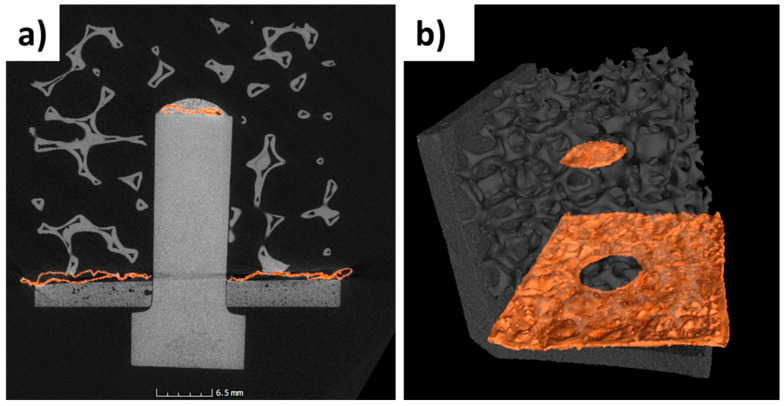
Tomographic reconstruction of the mock-up, showing the pin/substrate area (**a**) and the joining material (orange) at the bottom of the joined components (substrate and foam) and on the top of the pin (**b**).

Moreover, the computer-assisted tomographic analysis revealed that the joining material is confined to the interfacial area and does not flow in the foam open porosity. A slight misalignment between the axis of the pin and the foam (Figure 14a) can be detected. This joining process has been carried out without any sample holder, but ongoing activity will be addressed to jig manufacturing or a suitable assembly set up for a better alignment to keep in place all components during joining heat treatment.

At the moment, no information is available on the thermal conductivity of the proposed design, i.e., on the thermal performance of the joint. Results of the lab-scale thermophysical characterization of the All-SiC by LiqTech and porous SiSiC by Engicer are summarized in [26]: the thermal diffusivity, specific heat, and thermal conductivity are provided and compared with the literature data available.

## 4. Conclusions

The H2020 NEXTOWER project explored new SiC-based solutions for open volumetric air receivers; the goal of the experimental activity was to develop independently and simultaneously two designs to allow a comparison of all-SiC designs optimized for oxidation vs. the SiSiC design optimized for higher toughness and thermal conductivity. The following conclusions can be achieved derived from our investigations:

- With an optimal preparation of the extrusion and slip casting slurry and appropriate casting time, the all-SiC solar receiver can be manufactured with good green strength and wall thickness to be able to glue them together in green. The solid load of the extrusion slurry is 70–80 wt%, and from the slip casting is 60–70 wt% with a casting time of 10 min.
- For all-SiC components, the sintering of the monolith was studied for three different temperatures in an argon environment. It was found that the optimal preparation temperature to obtain a defect-free component with high mechanical strength (54 ± 1.3 MPa) ranges from 2000 °C to 2400 °C with a pore size in D50 of 17 µm.
- Ceramic thermal receivers for atmospheric air solar power towers have been successfully manufactured using optimized SiSiC lattices; a lattice geometry was designed, dimensioned, and printed through additive manufacturing.
- The SiSiC structure contains an optimized porosity gradient with the aim of increasing the heat exchange efficiency; the porosity of this structure ranges from 83% to 92% moving from the internal part to the external one; a thermal treatment was studied to reduce the reflected radiation on the final component.
- A joining process based on in situ formations of a MoSi_2_ particle reinforced Si.
- Matrix composite was found to be an effective method to join SiSiC based cups and foams for the thermal receiver.
- The joining method was also applied for the manufacturing of joined components, coupled to a mechanical fastener, in order to increase the mechanical resistance of the receiver and its durability.

In conclusion, innovative SiC ceramic materials for high-temperature open volumetric receivers based on (1) all-SiC honeycomb design for more durability to oxidation and (2) more flexible multi parts SiSiC 3D printed design for higher toughness, higher thermal conductivity, and more open design has been developed. A pressureless joining technique with proven scalability able to be coupled with mechanical fastening in order to avoid interfacial cracking of the solar receiver made of several pieces has been tested.

The next step in the framework of thermophysical characterization will be the investigation of the manufactured ceramic components under operative conditions.

As mentioned in the discussion section, solar thermal ageing tests of SiC volumetric receivers provided by Liqtech and EngiCer are in progress at the Plataforma Solar de Almería (PSA), Spain.

Further investigation will address the optimization of both SiC-based components in terms of morphology (porosity, foam wall thickness, etc.) and properties (thermal conductivity, mechanical strength) and joining process once the results of solar thermal ageing are available. Post-mortem analysis on tested samples will be carried out to improve the specific properties of the volumetric ceramic components.

## Data Availability

The data presented in this study are available on request from the corresponding author.
